# Decoding Individual differences and musical preference via music-induced movement

**DOI:** 10.1038/s41598-022-06466-3

**Published:** 2022-02-17

**Authors:** Yudhik Agrawal, Emily Carlson, Petri Toiviainen, Vinoo Alluri

**Affiliations:** 1 Cognitive Science Lab, International Institute of Information Technology, Hyderabad, India; 2 Department of Music, Art and Culture Studies, University of Jyväskylä, 40014 Jyväskylä, Finland

**Keywords:** Personality, Perception, Computational models, Machine learning

## Abstract

Movement is a universal response to music, with dance often taking place in social settings. Although previous work has suggested that socially relevant information, such as personality and gender, are encoded in dance movement, the generalizability of previous work is limited. The current study aims to decode dancers’ gender, personality traits, and music preference from music-induced movements. We propose a method that predicts such individual difference from free dance movements, and demonstrate the robustness of the proposed method by using two data sets collected using different musical stimuli. In addition, we introduce a novel measure to explore the relative importance of different joints in predicting individual differences. Results demonstrated near perfect classification of gender, and notably high prediction of personality and music preferences. Furthermore, learned models demonstrated generalizability across datasets highlighting the importance of certain joints in intrinsic movement patterns specific to individual differences. Results further support theories of embodied music cognition and the role of bodily movement in musical experiences by demonstrating the influence of gender, personality, and music preferences on embodied responses to heard music.

## Introduction

Humans appear to have a remarkably fine-tuned and robust ability to discern information about others based on bodily movement. For example, Troje, Westhoff and Lavrov^1^ showed that participants could easily learn to identify different individuals from point-light recordings of their gait, even scoring three-times above chance when recordings were rotated and manipulated to remove information about size and speed. In a follow up study, Westhoff and Troje^2^ additionally used a Fourier Transform to remove the most prominent frequencies from point-light stimuli, and found that identification was still above chance, and that participants were easily able to generalize information learned from different viewing angles. Being able to identify an individual from limited perceptual information has clear evolutionary advantages, particularly in the uniquely social context of early human cultures, where identifying group members and non-members could be necessary for survival^3^. Along these same lines, it could also be considered adaptive for other information, such as gender, mood state, or individual characteristics such as personality, to be encoded in and identifiable from a person’s bodily movement. Barclay and Cutting^4^ demonstrated that, on average, observation of just two step cycles was sufficient for participants to correctly identify gender from point-light displays while Koppensteiner^5^ has shown that even limited movement information from the head and hands can be used by observers to judge extraversion and neuroticism. Thoresen, Vuong and Atkinson^6^ found that observers made reliable judgements about personality from gait cues, although these judgements did not always align with the self-reported personalities of the walkers.

While it is not yet clear exactly which features of human movement allow for such information to be decoded, computational analysis of complex movement offers a way to explore how information about individual differences are encoded in subtle ways (e.g., how different joints move in relation to another in a particular dimension) that make it difficult to identify with the naked eye. Computational analysis of gait has been used to identify individuals^7^, to classify walkers according to gender^8,9^, and to identify individual differences of personality^10,11^ and emotion^12^.

Although gait is probably the most common means of studying individual characteristics as they relate to features of bodily movement, a paradigm in which participants perform free, spontaneous dance movements offers the potential advantage of greater individual variability of movement as well as theoretical connections to a range of psychological and social functions. Music and dance are found in every known human culture and play important roles in social contexts, and in most cultures represent largely inseparable phenomena^13^. Cross^14–16^ has suggested that music and dance, by virtue of their capacity to express non-specific but individually interpretable meaning, which he terms ’floating intentionality,’ may have played a role for early humans in negotiating times of intra- and inter-group uncertainty, such as the changes marked by wedding or coming-of-age ceremonies. Christensen, Cila-Conde and Gomila^17^ have proposed six different neural and bio-behavioral functions of human dance, including communication, self-intimation, and social cohesion.

A number of studies have explored perception of individual characteristics from dance movement. For example, Van Dycke et al.^18^ found that, when presented with side-by-side avatars of dancers expressing happiness or sadness, participants could correctly identify which emotion was being expressed. Much of the research into perception of dance movement has focused on gender, both in terms of the perception of gender from dance movement and, drawing on hypotheses that one of the evolutionary roles of dance has been sexual selection, the perception of attractiveness and other qualities related to mate-selection from dance. Pollick et al.^19^ have shown that gender can be accurately percevied from point-light displays of dance movement. Hufschmidt et al.^20^ found that both children and adults could accurately identify dancer gender from avatar movements, while Weege et al.^21^ found that female raters found the movements of male dancers with greater hand-grip strength to be more attractive. While it is not yet clear which movement features raters use to identify gender, differences in movement may arise from differences in average body structure and joint flexibility between genders. Fink et al.^22^ have hypothesized that, on an individual level, dance movements serve to signal information related to mate selection, while on a group level, dance movement serves such functions as coalition signaling and supporting group coordinated action.

Computational analysis of individual differences in dance movement has begun to provide insights into how such information is encoded in kinematic features. Carlson et al.^23^ found that individual movement signatures from participants moving spontaneously to music (i.e., as one might in a club or party setting), as captured by the three-dimensional co-variance of movement between joints, could be used to accurately classify individual dancers at a rate of 94%, suggesting that dance movements may be highly individualized. However, research has also shown correlations between dancers’ personality traits and features of their free dance movements, suggesting that such group-level individual differences are also encoded in dance movements. Luck et al.^24^ used principal component analysis to identify five components of free dance movement, showing that neuroticism, for example, was negatively correlated with global movement (movement of the whole body across the dance floor) but positively correlated with local movement (movement within the body), while Openness was positively correlated with local movement, and extraversion was positively correlated with all five components, suggesting extraverted participants tend to move more in general. Carlson, Burger and Toiviainen found that dancers’ self-reported trait empathy related to how much dancers changed their movement in response to different dance partners^25^. These studies suggest that it should be possible to predict participants’ personalities from their dance movements, although prediction (as opposed to correlation) has not yet been done.

Although Carlson et al.^23^ were able to identify individuals via dance movement with high accuracy, in the same study their attempt to classify which of the eight genres the participants were dancing to resulted in an accuracy of only 23 percent. One explanation for this may be that individual factors, such as personality and dancers’ preferences for some genres over others, had a greater influence on their movements than did the specific characteristics of the musical stimuli. Research into how music preference is embodied in dance, however, is limited. Luck et al.^26^ found that participants’ preferences for different excerpts resulted in a U-shaped curve on a number of kinematic features, suggesting that both low and high preference for particular musical excerpts resulted in more movement. However, further research is needed to explore the interaction of preference and movement on the level of musical genres, which, despite being a fuzzy concept^27^, are nevertheless a common way music preferences are discussed and related to individuals’ self-concepts and judgements of others^28,29^. Previous work additionally provides broad support for the existence of relationships between music preferences and individual differences, including personality trait empathy, and trait systemizing^28,30^. In light of the transitivity property which states that if A relates to B, and B relates to C, then A relates to C. In our case A refers to movement, B refers to Personality, and C refers to Music Preferences. If movement patterns have been associated with personality traits, and personality traits with music preferences, then we could assume that movement patterns would be associated with music preferences. A potential mechanism by which this may occur is that listening to music of a preferred genre leads to the recruitment of cognitive faculties related to greater attention to and emotional involvement with the music, which in turn results in potentially identifiable implicit movement patterns.

The current study aims to address these many potential influences on an individual’s music-induced movements, by using free dance movement to predict dancers’ gender, personality traits, and music preference. To this end, we use data sets from Carlson et al.^31^ and Luck et al.^24^ and employ machine learning techniques to predict Gender, Personality, and musical preferences from music-induced movement. We follow Carlson et al.^23^ in making use of co-variance between joints as a kinematic feature, as Troje, Westhoff and Lavrov^1^ and Westhoff and Troje^2^ have suggested that phase relationships between joints may have perceptual validity as a feature used in perceiving human movement. We aim to assess the robustness of our proposed machine learning architecture by comparing its accuracy on the two different data sets, allowing us to explore the degree to which findings generalize. Additionally, we attempt at proposing a *Joint Importance profiles* which is novel measure to evaluate the importance of specific joints and their relative movement in characterising personality and music preferences.

In light of previous studies, we investigate the following hypotheses:

- We expect, based on previous research showing that gender can accurately be classified from gait, that it will be possible to accurately classify gender from free dance movement as well.
- Since personality traits are stable, we can expect the findings to generalise to other datasets if we are able to capture them well enough for one. We also expect, based on previous research, that personality will be possible to predict from joint co-variance. Given that joint co-variance relates to local movement (as opposed to global movement across a dance floor), in light of findings from Luck et al.^24^, our predictions may be more accurate for neuroticism and less accurate for conscientiousness and extraversion as they were found to be positively correlated with global movement.
- Due to evidence of relationships between personality and musical preferences, we additionally expect that music preferences may also be predicted using participants’ free dance movements.

## Results

### Gender classification

The results for Gender classification on Dataset-1 and Dataset-2 can be found in Table 1.

**Table 1:** Gender classification results using position data and velocity data for five personality traits using SVM classification on both the datasets.

Classification accuracy (in %)	Dataset-1	Dataset-2
Position	96.53	98.76
Velocity	84.59	86.33

As can been seen from Table 1, clearly position data gives a higher accuracy than velocity data for both the datasets. We achieved slightly higher accuracy for the second dataset, which can be attributed to the fact that Dataset-2 is almost twice as big compared to Dataset-1.

### Personality prediction and joint importance

Overall, Bayesian Regression demonstrated superior performance over Principal Component Regression, hence we only report those results. Moreover, Bayesian Regression provides confidence bounds for our prediction which enable us to evaluate the uncertainty of the predictions. The results for personality prediction on Dataset-1 and Dataset-2 using Bayesian Regression can be found in Table 2. The results using Principal Component Regression can be found in supplementary material. Moreover, using position data as input features provided superior prediction when compares to velocity data. Hence we report here detailed results of Bayesian Regression based on position data.

**Table 2:** Prediction results using position data for five personality traits using bayesian regression on both the datasets.

	Openness		Conscientiousness		Extraversion		Agreeableness		Neuroticism	
	RMSE	*R*^2^	RMSE	*R*^2^	RMSE	*R*^2^	RMSE	*R*^2^	RMSE	*R*^2^
Dataset-1	0.20	0.78	0.32	0.76	0.38	0.74	0.25	0.78	0.38	0.76
Dataset-2	0.19	0.90	0.22	0.90	0.25	0.88	0.17	0.89	0.24	0.88

Bayesian Regression performed considerably well for both the data sets as evidenced by the high proportion of variance explained, that is, average *R*^2^ score of 76.3% and 89.0% across all traits for Dataset-1 and Dataset-2 respectively using the position data. On the other hand, using velocity data resulted in a lower average *R*^2^ score of 44.4% and 39.0% for Dataset-1 and Dataset-2 respectively. Furthermore, the considerably low RMSE values when compared to the range of personality values (i.e., 1.0–5.0) reflects high model accuracy. The average RMSE scores for Dataset-1 and Dataset-2 using the position data was found out to be 0.31 and 0.21 respectively, which was considerably smaller than the avg. RMSE scores of 0.47 and 0.53 using the velocity data for Dataset-1 and Dataset-2 respectively.

**Figure 1:**
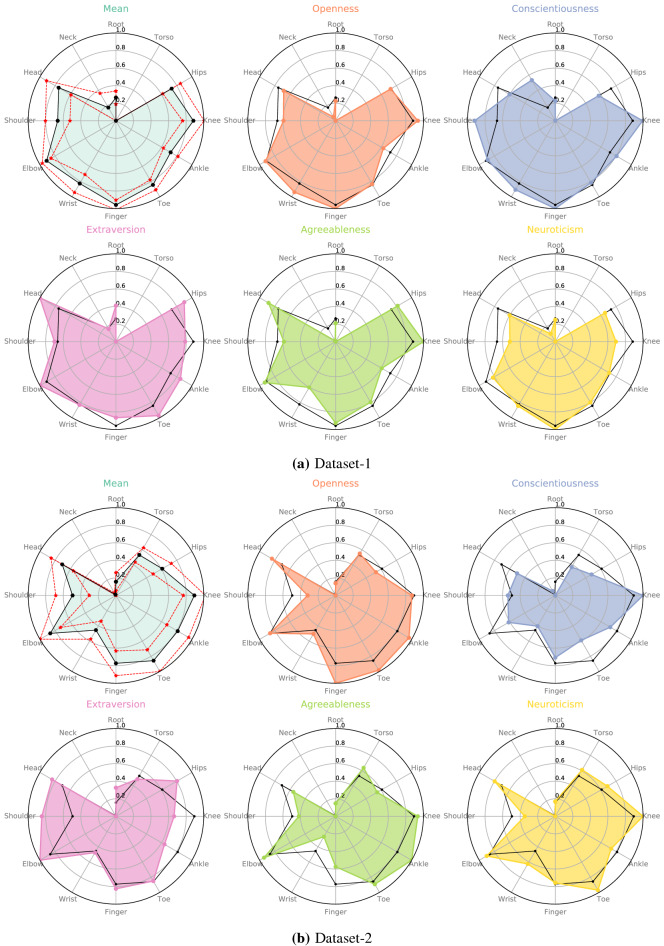
Relative importance of Joints of the five personality traits (Openness, Conscientiousness, Extraversion, Agreeableness, and Neuroticism) using the Position Data. The black line indicates the mean importance of the corresponding joint marker. The red dotted line in the top left sub-figure indicates the standard deviation about the mean.

For evaluating *Joint Importance* we used learned weights of the model using position data across the different prediction tasks. In order to get an overview of the relative importance of joints, we averaged those joints which occur in pairs (e.g., L and R shoulder, L and R knees, etc.), thus reducing the total number of joints to 12. This is referred to as the *Joint Importance profile*. Figure 1a,b display relative personality-wise *Joint Importance* for Dataset-1 and Dataset-2 respectively. The black line plotted in each sub-figure indicates the mean of *Joint Importance* across personality traits for the respective data set. The farther away from the mean the *Joint Importance* value is, the more important that joint is in characterizing that trait.

Altogether the results characterizing an individual personality trait is dominated by the limbs than the core of the body. As seen from the Fig. 1a,b, Extraversion and Conscientiousness show some similarity across the datasets, reflecting the importance of specific joints. This is reflected in the Spearman correlation between the *Joint Importance profiles* across datasets: only Conscientiousness exhibited significant correlation ($r =.73$, $p < .01$) while Extraversion demonstrated borderline significance ($r = .52, p = .08$). For Extraversion, the ’Head’, ’Hips’, ’Shoulder’, ’Elbow’, and ’Knee’ are consistently more important across datasets. Similarly for Conscientiousness, the ’Head’, ’Shoulder’, and ’Knee’ are consistently more important.

### Generalization of trait-wise movement patterns

**Figure 2:**
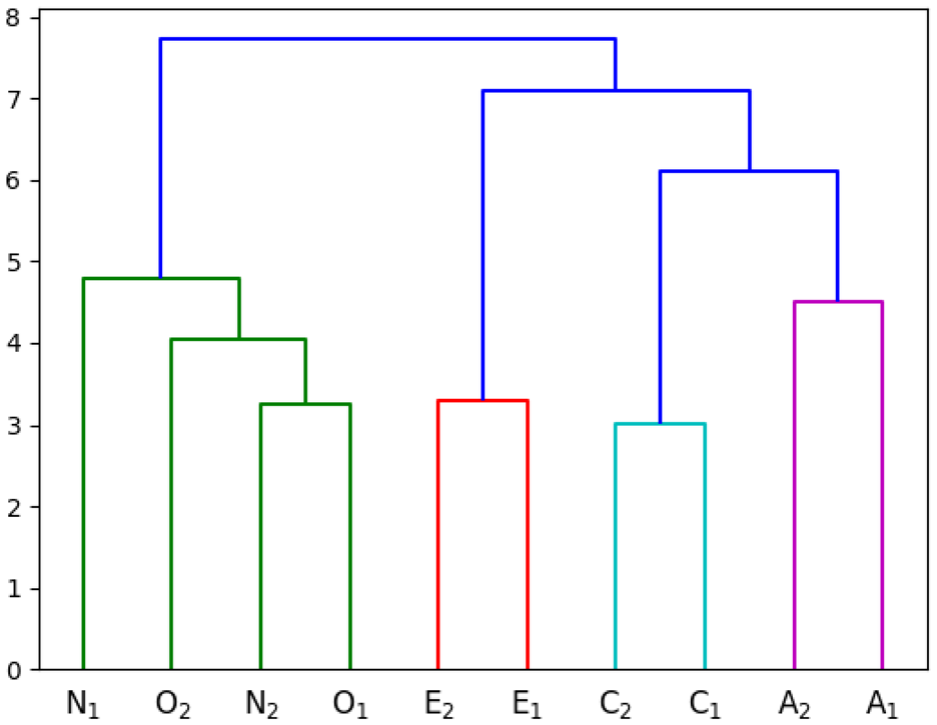
Dendrogram of the learned *Joint Importance profiles* for Personality traits in different datasets. Number {1, 2} in the subscript denotes the dataset number. Each color represents cluster at some level.

To investigate consistency of movement patterns for individual traits across data sets, we perform Agglomerative Hierarchical clustering on the *Joint Importance profiles*. Agglomerative Hierarchical clustering allows to cluster the profiles in a hierarchical manner by capturing inherent similarities. We apply the commonly used ward’s linkage method to compute the distance between the clusters, as it tends to produce homogeneous cluster and yields compact spherical clusters when compared to other approaches^32^. Figure 2 shows the dendrogram of the learned *Joint Importance profiles* of different personality traits across data sets.

**Figure 3:**
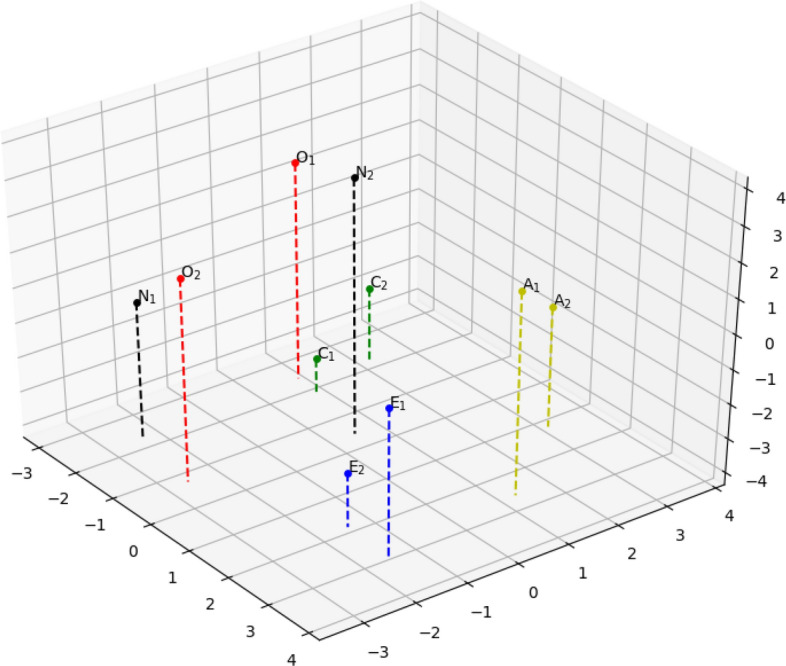
Multi-dimensional scaling (MDS) results for the learned *joint importance profiles* for personality traits on both the data sets. Number {1, 2} in the subscript denotes the dataset number.

As seen in Fig. 2, traits Extraversion, Conscientiousness, and Agreeableness, demonstrate high inherent similarity in *Joint Importance profiles* suggesting that individual prediction models are similar. In other words, this implies that there exist trait-specific movement patterns. On the other hand, Openness and Neuroticism cluster together suggesting similarities in their overall movement patterns. However, agglomerative clustering only reveals similarities at a cluster level but not how each of the elements within a cluster relate to each other. Hence, we perform Multidimensional Scaling (MDS), a common technique that is used to visualize interrelationships within high-dimensional data in a lower dimensional space. We perform MDS by creating the dissimilarity matrix based on the Euclidean distance between the *Joint Importance profiles* and projecting them onto a 3-dimensional space. Figure 3 provides the 3D representation of *Joint Importance profiles* similarity as a result of MDS revealing high trait-wise similarity across data sets.

### Prediction of music preference

As was the case in personality prediction models, Bayesian Regression outperformed Principal Component Regression; thus, we report the results using Bayesian Regression. In addition, using position data as input features provided superior prediction when compares to velocity data. The results of music preference prediction using Bayesian regression for Dataset-1, with position data as input features, can be found in Table 3.

**Table 3:** Prediction results using position data for twelve STOMP based musical preference (Blues, Country, Dance, Funk, Jazz, Metal, Oldies, Pop, Rap, Reggae, Rock, and Soul) using bayesian regression on Dataset-1.

Metric	Blues	Country	Dance	Funk	Jazz	Metal	Oldies	Pop	Rap	Reggae	Rock	Soul
*R*^2^	0.81	0.73	0.84	0.72	0.77	0.84	0.75	0.85	0.72	0.73	0.79	0.77
RMSE	0.58	0.96	0.63	0.65	0.72	0.85	0.63	0.53	0.94	0.81	0.49	0.72

From Table 3, we can see that the Bayesian regression model was able to predict musical preferences with high accuracy. The range for *R*^2^ score varies between 72% and 85% for different genres with an avg. *R*^2^ score of 77.5% using the position data. Additionally, the low RMSE values ranging from 0.49 to 0.96 when compared to the range of self-reported music preference values (i.e., 1.0–7.0) demonstrates high model precision.

**Figure 4:**
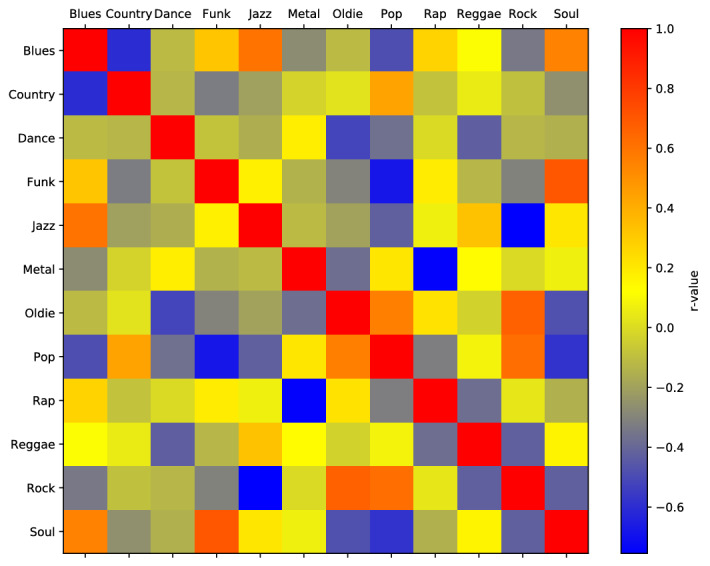
Spearman correlations between *Joint importance profiles* for different Musical Preferences.

Figure 4 displays Spearman Correlations between *Joint Importance profiles* that contribute to the prediction of Musical Preferences. Based on our hypothesis, we expected to see similar *Joint Importance profiles* for genres that are jointly preferred per trait. As can be seen in Fig. 4, highest positive Spearman correlation was observed between the Joint Importance profiles of Jazz and Blues (r = 0.60), Soul and Funk (r = .7), Pop and Rock (r = 0.62), and Rock and Oldie (r = 0.66), supporting our hypothesis. Additionally, we also observe highest negative correlations between the Joint Importance profiles of Rock and Jazz (r = − 0.75), and Metal and Rap (r = − 0.75), Funk and Pop (r = − 0.68), Blues and Country (r = − .6), and Pop and Soul (r = − 0.58).

**Figure 5:**
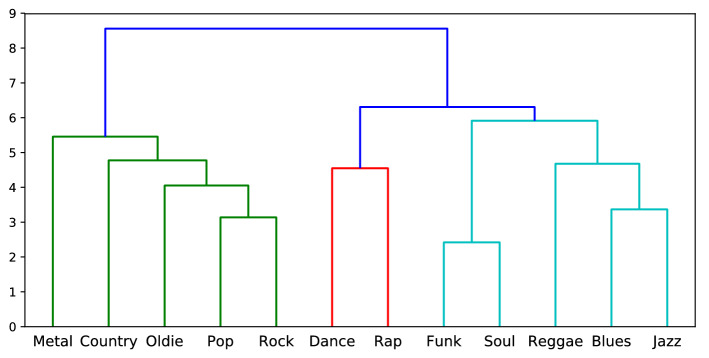
Dendrogram for *Joint importance profiles* across STOMP music preferences.

In order to better capture the intrinsic similarities in *Joint Importance profiles*, we performed agglomerative hierarchical clustering on the learned *Joint importance profiles* for different genres. The clustering was performed using the same approach as explained in the section above. Figure 5 represents the dendrogram of hierarchical clustering on the *Joint Importance profiles*.

We observe from Fig. 5, the similar genres such as Funk & Soul, and Blues & Jazz, cluster together early in the hierarchy tree. This clustering pattern reveals similar genres being merged in the beginning suggesting similar overall movement patterns for those genres.

**Figure 6:**
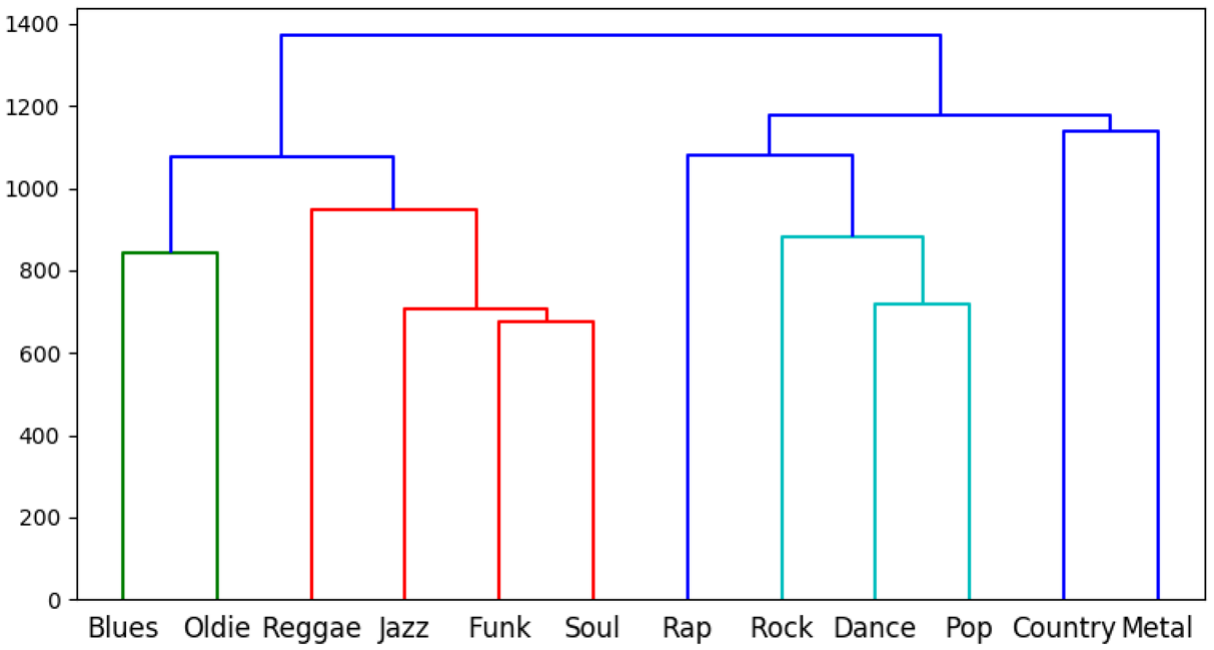
Dendrogram of the self-reported ratings for STOMP music preferences.

We further compare the similarities between the self-reported participants’ ratings of the Music preferences with learned *Joint Importance profiles*. Figure 6 represents the dendrogram of hierarchical clustering on the self-reported ratings. Comparing this with Fig. 5, we see that self-reported ratings revealed an intrinsic structure fairly similar to those of movement patterns. In particular, we find two broad clusters. In the first cluster, we observe that similar clustering for the genres Funk and Soul, and Blues, Jazz and Reggae. In the other cluster, the genres Rock, Pop, Metal and Country exhibited high similarity. The one exception was that of the genres Dance and Rap showing high similarity in *Joint Importance profiles* but father away in terms of user preference.

## Discussion

Our results showed that we could successfully predict dancers’ gender, personality traits, and music preferences from their music-induced movement. Our model was able to classify gender with near perfect accuracy/high precision and the regression model was able to predict personality and music preferences with high accuracy. These results provide broad support for the influence of gender, personality, and music preferences on embodied responses to heard music, corroborating ideas of embodied music cognition and the role of bodily movement in musical experiences. These results provide support for the hypothesis that socially relevant information is encoded in dance movement, in line with previous research showing human ability to perceptually decode personality and gender from bodily movement. The encoding of multiple types of socially relevant information in dance movements makes sense in light of the fact that dance typically occurs in social settings and often in contexts which include strangers as well as acquaintances (e.g., a wedding, party, or night club). In such settings, the ability to decode such information on the bases of limited, embodied interactions might be adaptive as it allows some initial social judgements to be made quickly without the need for conversation or extended behavioral interactions.

In addition to the above findings, we devised a novel measure to evaluate the importance of specific joints and their relative movement in characterizing particular personality traits and music preferences. This measure allows us to better understand how socially relevant information is encoded in dance with a degree of detail that is unlikely to be available to conscious observation; that is, we may be able to perceive personality or trait empathy based on how another person moves, but are unlikely to be able to consciously identify all of the relevant movement features which contribute to our impression. Our results are the first to our knowledge to demonstrate the generalization of an analytical architecture across two dance-movement data sets collected using two different sets of musical stimuli. Although neither data set used in our analysis is large enough to make a commercial or industrial grade model, we were able to utilize two data sets of modest size to expand theoretical understanding and provide support for the robustness of our computational model. It is notable that neither data set was collected with the express purpose of allowing for such information to be decoded. While some bias may be introduced due to the fact that both data sets were collected in the same location (albeit several years apart), both were also collecting using a naturalistic paradigm, giving us reason to be optimistic that at least some aspects of the current results may generalize further.

As hypothesized, we were able to predict gender with near perfect accuracy of 96.53% and 98.76% for Dataset-1 and Dataset-2 respectively. As Troje^33^ was able to achieve high accuracy of gender minimal information, such as a single eigenpostures or as few as four principal components, it is not surprising that we were able to achieve such high accuracy. Identifying gender with such high accuracy from music-induced movement suggests that there are gender specific movements to music. These gender differences may be the result of sociocultural influences as well as differences in natural movement patterns influenced by gender-specific joint flexibility.

Our Bayesian regression model for prediction of personality demonstrated an average *R*^2^ score of 76.3% and 89.0% across all traits for Dataset-1 and Dataset-2 respectively using the position data. Previous studies have shown Extraversion to be positively related to global movement as opposed to Neuroticism which relates negatively to global movement^24^. As hypothesized, given that joint co-variance is related to local movement, Neuroticism was found to have slightly better classification accuracy than Extraversion. The current findings are especially interesting in light of the findings of Carlson et al.^23^, who used covariance matrices derived from motion capture data in an analysis in which individual dancers were correctly classified using SVM classification with an accuracy of over 80 percent, suggesting that covariance between markers encodes individual differences both on the level of individual identity and on group-level features such as personality.

The proposed novel measure to evaluate the importance of specific joints and their relative movement in characterizing individual traits demonstrated that the limbs of the body to have more significance in predicting individual traits than the core body. The ’Finger,’ ’Elbow,’ and ’Knees’ were found to have the greater impact on *Joint Importance*, while the ’Root,’ ’Neck,’ and ’Torso’ had an insignificant impact. This is consistent with the fact that gestures are necessary for communication^34^. Extraversion and Conscientiousness have some similarities in terms of *Joint Importance profiles* across data sets, indicating the importance of specific joints. ’Head, Shoulder, and Knee’ play a crucial role in characterizing Conscientiousness, and these joints were consistently more important across data sets. For Extraversion, the ’Head,’ ’Hips,’ ’Shoulder,’ ’Elbow,’ and ’Knee’ are consistently more important across the data sets. Luck et al.^24^ discovered a link between Extraversion and head movement speed, confirming the current theory that the head is particularly important in determining Extraversion. Carlson et al.^35^ found that in relation to Extraversion, the core body was more significant in responsiveness to musical tempo than Conscientiousness, which is partly confirmed by the marginally higher importance of the finger and wrist markers in our analysis but partly refuted by the importance of the shoulder marker in Extraversion. Overall, the disparity in results may be due to the current study’s use of position data rather than velocity or acceleration data. As a result, the specific markers relevant to predicting individual traits sometimes contradict and largely corroborate previous research.

Agglomerative clustering and dendrogram plots of the *Joint Importance profiles* for personality provided evidence for the ability of our regression model to generalize across data sets by demonstrating that the weights learned by different models are similar. We have further shown the generalization by visualizing the learned profiles using Multi-Dimensional Scaling (MDS) based on Euclidean distance. The five mini clusters formed for each personality trait from the different data sets provides further corroboration that the weights learned by different model for two data sets are similar. As hypothesized, we were able to successfully predict the personality values for the second data set, which further demonstrates the robustness of our model. We were able to achieve higher accuracy for the second data set, which can be attributed to that set’s larger sample size. While we did not use a single model to train and evaluate the different data sets, due to the dissimilarities of genres and participant demography, our model nevertheless showed similarities between the two data sets.

Related to prediction of STOMP-based Music Preferences, our regression model performed considerably well, demonstrating an average *R*^2^ score of 77.5% with the score varying from 71.9% for Funk to 85.3% for Pop at the higher side for Music Preferences. To a great extent, the *Joint Importance profile* learned from the model using the position data and participants’ self-reported ratings of music preference show similar clustering pattern. From Figs. 5 and 6 it is clearly visible that music preferences like Funk and Soul, and Blues, Jazz and Reggae are clustered similarly in both dendrograms in one of the higher-level clusters. In the another higher-level cluster, there were also similar clustering between Rock, Pop, Metal and Country, as well as between Dance and Rap. This is a notable finding, as self-reported music preferences are made consciously using verbal representation, which Leman^36^ has argued are problematically distant from direct (embodied) engagement with music. That we could find similarities in clustering between reported preferences and movement patterns suggests that, despite being at a far remove from conscious, verbal descriptions, embodied responses to specific, heard music can indeed be seen to encode music preferences at the genre level.

Another observation related to this point from Fig. 5 is that Oldie and Country both belong to the “Upbeat and Conventional” factor as suggested by Rentfrow and Gosling^28^, and clustering via *Joint Importance* represents a closer relationship for these compared to self-reported ratings; that is, in this case, movement patterns were more closely associated with Rentfrow and Gosling’s model than were verbal self-reports. This may be due to similarity of movement in response to acoustic similarities between these genres. This result may also reflect differences in participants’ perceptions of genre compared to industry-standard labeling, which have been shown to be ambiguous and inconsistent, as well as subject to cultural differences in the distinction between more granular categories^27^. Overall, these results indicate a relationship between embodied responses to music to specific stimuli and more general music preferences that is detectable despite the many other factors that may have influenced dancers’ movements, including specific musical features. Taken together with our results regarding personality, this analysis demonstrates that abstract psychological and psychosocial concepts such as personality and preference are indeed evidenced by concrete features of complex dance movement, corroborating the idea of dance as a useful paradigm in exploring how socially relevant information is encoded in human movement.

The achieved prediction accuracy using covariance of position data as input features was about twice as high when compared to features extracted from velocity data. One possible explanation is that position data captures individual bodily structure and differences in joint flexibility which might give rise to certain kinds of movement patterns. The methods developed in the current study provide a functional basis from which to continue this work.

To conclude, this study represents an early step to making this approach applicable to personalized gesture-based retrieval systems. With recent development in the field of 3D human pose prediction, which can predict the human body joint coordinates from a monocular video, it can be applied to monocular video taken by devices such as a cell phone camera. This would then allow future recommendation systems to take embodied processes into account, resulting in better and more responsive personalized experiences. Future research could help clarify the relationship between the current results and those movement features which are perceptually relevant to judgements of gender, personality and music preference. While analysis showed interpretable relationships within and between sets of *Joint Importance profiles*, further research is needed to determine whether, how, and in what contexts this encoded information may be decoded by human observers. Such research may have particular relevance to our understanding of social perception of human movement, and may further understanding and treatment of disorders involving social deficits, such as autism^19,37^.

## Methods

### Data sets

We used two data sets in our study. They comprised spontaneous movement data of participants moving to musical stimuli representing different genres. The data were obtained via Motion Capture systems. A brief overview of both the data sets is provided in Table 4.

**Table 4:** Dataset comparison.

	Dataset-1	Datatset-2
Participants	58	60
Gender	41 Females, 17 Males	43 Females, 17 Males
Age	Mean: 26.8 years, Std: 4.7 years	Mean: 24 years, Std: 3.3 years
Personality (BFI)	✓	✓
Music preferences (STOMP-R)	✓	✗

#### Dataset-1

The first dataset is from the study by Carlson et al.^31^ comprising data from 58 university students (41 females; mean age = 26.8 years, std = 4.7 years). Participant gave written consent and participation was completely voluntary. All experiments were performed in accordance to the guidelines and regulations of the National Advisory Board on Research Ethics in Finland (TENK). Ethical permission was not needed for this kind of research, according to the aforementioned guidelines and regulations.

Thirty-six individuals reported having received formal musical training, while twenty participants reported having received formal dance training. The stimuli comprised sixteen 35-second excerpts from eight genres, in randomized order: Blues, Country, Dance, Jazz, Metal, Pop, Rap, and Reggae. Participants were asked to listen to the music and move as freely as they desired that felt natural with regards to the stimuli presented. Additionally, participants were encouraged to dance if they wanted to, but staying within the marked capture space. The aim of these instructions was to create a naturalistic setting, such that participants would feel free to behave as they might in a real-world situation.

**Figure 7:**
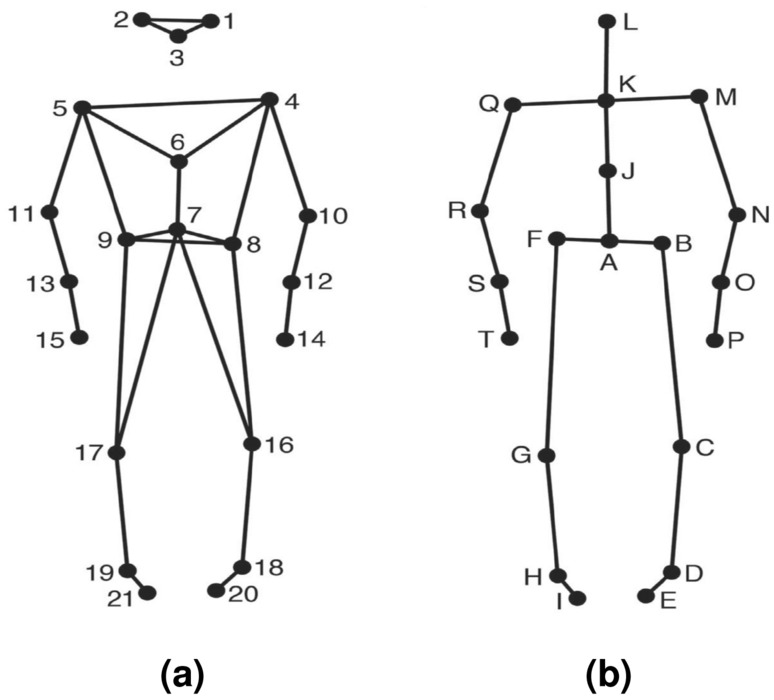
Dataset-1: Marker and joint locations (**a**) Anterior view of the marker locations a stick figure illustration; (**b**) Anterior view of the locations of the secondary markers/joints used in animation and analysis of the data.

Participants’ movements were recorded using a twelve-camera optical motion-capture system (Qualisys Oqus 5+), tracking at a frame rate of 120 Hz, the three-dimensional position of 21 reflective markers attached to each participant (see Fig. 7a). The MATLAB Motion Capture (MoCap) Toolbox^38^ was used to analyze this data. Data were first trimmed to the duration of each stimulus and, Following this, to simplify analysis and reduce redundancy, the original markers were transformed to yield a secondary set of markers subsequently referred to as joints, the locations of which are depicted in Fig. 7b.

The locations of joints B, C, D, E, F, G, H, I, M, N, O, P, Q, R, S, and T are identical to the locations of one of the original markers, while the locations of the remaining joints were obtained by averaging the locations of two or more markers; Joint A: midpoint of the two back hip markers; J: midpoint the shoulder and hip markers; K: midpoint of shoulder markers; and L: midpoint of the three head markers. The data were then transformed to a local coordinate system, in which the location of each joint was expressed in relation to the root joint (Fig. 7b, marker A), which is defined as the origin, and the line connecting the hip markers as the mediolateral axis, allowing for comparison between dancers regardless of their orientation within the original mocap space. Further, using the MoCap Toolbox, the instantaneous velocity of each marker in each direction was calculated by time differentiation followed by the application of a 2nd-order Butterworth filter with a cutoff frequency of 24Hz.

#### Dataset-2

For the second data set, sixty-four participants took part in the motion capture data collection in a study by Luck et al.^24^. Four participants were excluded from further analysis due to incomplete data. Thus, 60 participants were retained (43 females; mean age = 24 years, std = 3.3 years). They were recruited based on a database of 952 individuals that contained their scores of the Big Five Inventory^39^. The aim of the original study involved recruiting high- and low-scoring individuals for each of the five dimensions. Six participants had received formal music education, while four participants had a formal background in dance. All participants gave their informed consent prior to their inclusion in the study and were free to discontinue the experiment at any point. Ethical permission for this study was not needed, according to the guidelines stated by the University of Jyväskylä’s (Finland) ethical board.

Participants were presented with 30 randomly ordered musical stimuli. Among the 30 musical stimuli, five stimuli belonged to each of the following popular music genres: Jazz, Latin, Techno, Funk, Pop, and Rock. All stimuli were 30 seconds long, non-vocal, and in 4/4 time, but differed in their rhythmic complexity, pulse clarity, and tempo. As described for Dataset-1, the participants were directed to move freely to the music.

**Figure 8:**
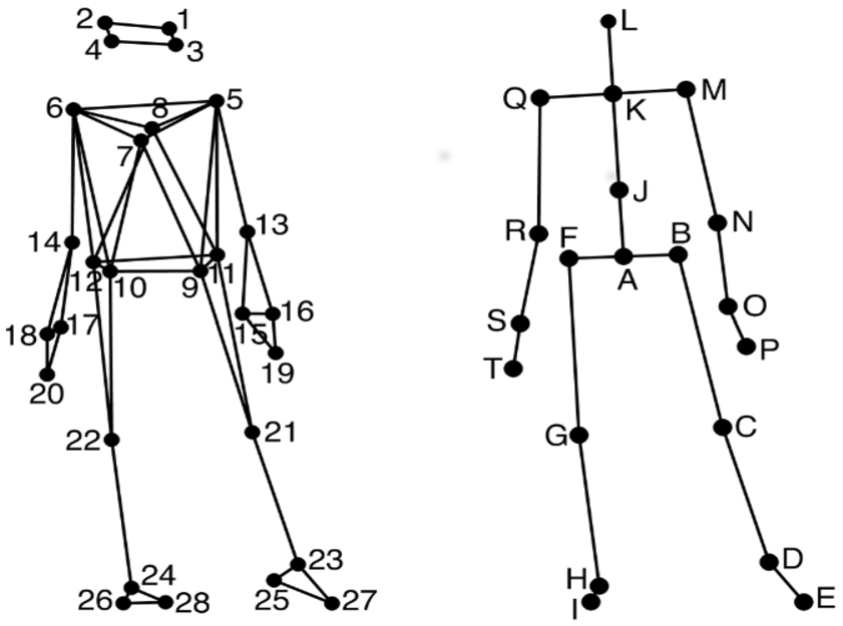
Dataset-2: Anterior view of the location of the markers attached to the participants’ bodies; Anterior view of the locations of the secondary markers/joints used in the analysis.

A similar process was followed as described in section Dataset-1 to transform the the 28-marker data into a set of 20 secondary markers, referred to hereafter as joints, displayed in Fig. 8. The locations of joints C, D, E, G, H, I, M, N, P, Q, R, and T are identical to the locations of one of the original markers, and the locations of the remaining joints were obtained by averaging the locations of two or more markers. Data were trimmed and transformed to a local coordinate system. The kinematic variables, position and velocity were estimated using the Savitzky-Golay smoothing FIR filter^40^ with a window length of seven samples and a polynomial order of two. These values were found to provide an optimal combination of precision and smoothness in the time derivatives.

#### Personality and music preferences measure

The Big Five Inventory (BFI) was used to capture the five personality dimensions, namely, Openness, Conscientiousness, Extraversion, Agreeableness, and Neuroticism^41^. This data is available for both the data sets. Music Preferences for the participants were measured for the Dataset-1 using a revised and updated version of the “Short Test Of Music Preferences” (STOMP)^28^, that is, the STOMP-Revised (STOMP-R)^42^. This version includes genres not found in the original STOMP, thus providing a broader initial pool of genres from which to choose. Genres that were not suitable for dancing (e.g., Classical, Opera) were eliminated. After careful considerations regarding the “Danceability” of stimuli belonging to genres and several other factors^43^ the total of 48 tracks from 12 genres were considered in the online listening experiment. Participants rated their liking for the heard stimuli on a seven-point Likert scale. Participants could listen to an excerpt more than once. After rating all 48 excerpts, participants then completed a version of the STOMP-R including only the 12 genres used in the experiment.

### Movement features

**Figure 9:**
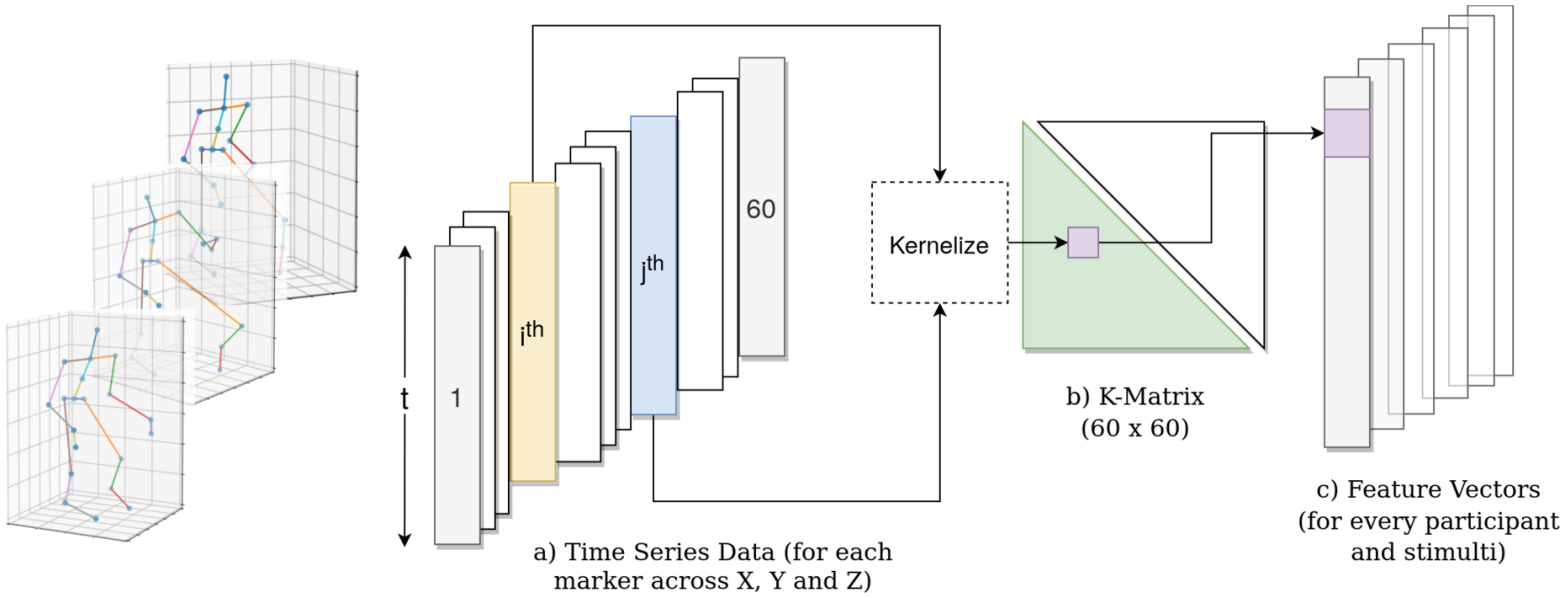
Overview of our feature extraction pipeline. Given the position of joints across time frames in 3D Euclidean space (**a**), we apply pairwise correntropy between time series $x_i$ and $x_j$ and calculate the K-matrix (**b**). Then, taking the lower triangular part of the symmetric covariance matrix, we get the feature vectors (**c**).

The pipeline for movement feature extraction is illustrated in Fig. 9. Using the MoCap Toolbox^38^, we derived the position and instantaneous velocity for the set of 20 markers as specified in sections Dataset-1 and Dataset-2. We calculated the marker by marker covariance matrix for each participant, for each of the stimuli, for position and velocity data separately. The covariance between all the marker time series data (position or instantaneous velocity) in each direction (X, Y, and Z) for each stimuli measures the degree to which the movement of any two markers in any direction covaried with each other across the entire stimulus. We used correntropy^44^, a non-linear measure to calculate covariance between the marker time series $x_{i}$ and $x_{j}$, given by:

1 $$K(x_{i} ,x_{j} ) = e^{{\frac{{ - \left\| {x_{i} - x_{j} } \right\|_{2}^{2} }}{{2\sigma ^{2} T^{2} }}}}$$

where $||x_{i} - x_{j}||_{2}$ is the L2-norm between $x_{i}$ and $x_{j}$, $\sigma$ is a constant set at 12.0 (based on^23^), and *T* is the length of the time-series. The L2-norm is normalized according to the length of the time-series to take account of different lengths of stimuli. Since the number of joints is 20 and each joint has three coordinates, the dimension of the covariance matrix *K* turns out to be $60 \times 60$. Owing to the symmetric property of *K*, the lower triangular part excluding the diagonal elements was vectorised to produce a feature vector of length 1770 for each participant and for each stimulus. The features extracted for Position and Velocity data were used to train the classification and regression models.

### Classifying gender from movement

In order to identify gender from movement, we used Linear Support Vector Machines (SVM), a classification technique that identifies the optimal solution for separating the classes in some hyperspace. SVM creates the largest possible buffer space between the two classes which is defined as the Optimal Separating Hyperplane, or OSH. The OSH minimises the risk of incorrectly classifying any new data^45^. The Euclidean Distance or L2-norm was used as a penalty measure to identify the optimal class boundary. The tolerance of $1e^{-5}$ was used as the stopping criteria.

### Predicting individual differences from movement

In order to predict personality and STOMP-related music preferences from movement patterns we used Bayesian Regression and Principal Component Regression (PCR). In Bayesian Regression the weights are treated as random variables belonging to an underlying distribution. By comparison, Linear Regression uses fixed weights. The predictions generated by Bayesian regression are not estimated to be a single value, but are assumed to be drawn from a probability distribution. Depending on the data set and its size, we can be more or less certain about the model weights. Thus, the predictions of the model also belong to a distribution, providing confidence bounds for our predictions, allowing a better representation of the uncertainty of a model’s predictions. PCR, on the other hand, is similar to linear regression with the independent variables of predictors represented as linear combinations of feature vectors.

We compared the aforementioned models and Bayesian Regression was superior so we only report results with that. Moreover, Bayesian Regression provides confidence bounds for our prediction which enable us to evaluate the uncertainty of the predictions. We used the Python based scipy toolkit^46^ to perform our analyses.

To evaluate the accuracy of the models, we use variance explained (i.e., $R^2$ ) and Root-mean-square-error (RMSE) as performance measures. RMSE estimates the deviation of an observed value from our model’s prediction, and has the useful property of being in the same units as the variable being predicted. The $R^2$ is a measure of variance explained in the data and represents goodness-of-fit.

RMSE and $R^2$ are estimated as an average of 5-fold cross-validation. To this end, we split the data randomly in 5 equal chunks, where each chunk is used as a testing set at some point. In the first iteration of 5-fold cross-validation, the first chunk (that is, 20 percent of total data) was used to evaluate the model while the remaining 80 percent of the data was used to train the model. This process was repeated 5 times such that all data was used at least once as the testing set. This process helps in obtaining a more generalized estimate of classification accuracy.

### Key movement patterns associated with predicting individual differences

**Figure 10:**
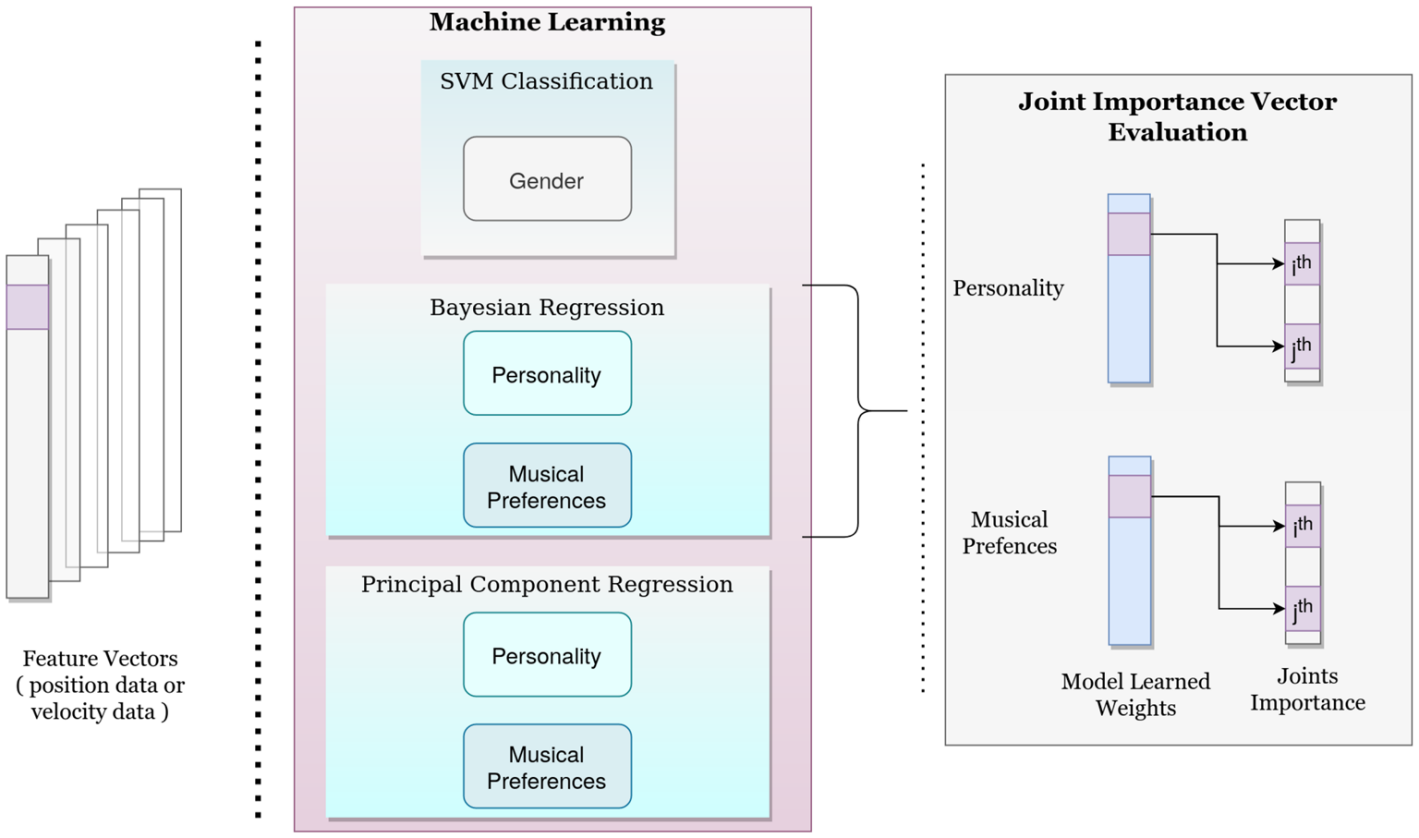
Overview of our ML tasks and *Joint importance vector* evaluation pipeline. Given the extracted feature vectors, we apply SVM for gender classification, and Bayesian Regression & PCR for predicting personality and musical preferences. Then, using the trained regression model, we evaluate trait-wise *Joint Importance vectors*.

The prediction pipeline and *Joint Importance Vector* evaluation is illustrated in Fig. 10. In order to identify the key movement patterns associated with predicting individual differences, we propose a novel *Joint Importance* metric. *Joint Importance* is a measure of the contribution of each of the joints in predicting each class (e.g., how important the shoulder or elbow joint is in predicting a given personality trait or genre preference). In the training phase, for each iteration of the 5-fold cross-validation, we obtain a trained model with weights associated with each of the 1770 input feature vector as mentioned above. Each of these 1770 elements is associated with pairwise correlation of joints in each of the three dimensions in Euclidean space. We first map the 1770 weights to the respective joint pairs. Then, for each joint, we find the joint importance by summing the absolute weights of those associated with the respective joint across all the 5 iterations. We sum the absolute weights because the magnitude preserves the importance of that joint-pair correlation. This process results in the *Joint Importance vector* of length 20. In order to visualize and compare *Joint Importance* across classes, we perform Min-Max Normalization. Min-Max Normalization is a standard procedure where the minimum value is transformed to 0, while the maximum gets set to 1. The pseudo-code to get the *Joint Importance* is provided in supplementary material.

## Supplementary Information

Supplementary Information.

## Data Availability

The datasets generated during and/or analysed during the current study are available from the corresponding author on reasonable request.
