# Supplementary material for: Decoding Individual differences and musical preference via music-induced movement
Source: Sci Rep. 2022 Feb 17;12:2672. doi: 10.1038/s41598-022-06466-3 (PMC8854731; doi:10.1038/s41598-022-06466-3)
Supplement: Supplementary file 1 — Supplementary Information. [file 41598_2022_6466_MOESM1_ESM.pdf]

Supplementary Information for:
“Decoding Individual differences and musical preference via music-induced movement"

Yudhik Agrawal, Emily Carlson, Petri Toiviainen, Vinoo Alluri

Calculating Joint Importance Vector from the learned weight vectors

Algorithm 1 Evaluating Joint Importance

Result: Calculate a vector J of 20 dimensions representing importance of each joint.

W is the weight vector; J is the importance vector initialised with 0; S contains lower triangular indices excluding diagonal indices; 0-indexing is followed;

$S \leftarrow LowerTriangularIndices(60 \times 60)$
$N \leftarrow S.length()$
for $k = 0 : N-1$ **do**
    $(i, j) := S(k)$
    $(\hat{i}, \hat{j}) \leftarrow IndexToJoint(i, j)$
    $J(\hat{i}) := J(\hat{i}) + |W(k)|$
    $J(\hat{j}) := J(\hat{j}) + |W(k)|$
end for
return J

Min-Max Normalization for Joint Importance Vector

$$\overline{JI}[i] = \left(\frac{JI[i] - min(JI)}{max(JI) - min(JI)} \right) \forall JI[i] \tag{1}$$

Evaluation Metrics

(a) Root-Mean Square Error (RMSE): It is given by

$$RMSE(y, \hat{y}) = \sqrt{\frac{1}{n} \sum_{i=1}^{n} (y_i - \hat{y}_i)^2} \tag{2}$$

where $\hat{y}_i$ is the predicted value of the ith sample and y_i is the corresponding true value for total n samples. (b) R^2 Score: the estimated R^2 is defined as:

$$R^2(y, \hat{y}) = 1 - \frac{\sum_{i=1}^{n} (y_i - \hat{y}_i)^2}{\sum_{i=1}^{n} (y_i - \bar{y})^2} \tag{3}$$

where $\hat{y}_i$ is the predicted value of the ith sample, y_i is the corresponding true value for total n samples, and $\bar{y}$ is the mean of the ground truth data.

Principal Component Regression results for Personality Prediction

Principal Component Regression		Openness		Conscientiousness		Extraversion		Agreeableness		Neuroticism	
		RMSE	R2	RMSE	R2	RMSE	R2	RMSE	R2	RMSE	R2
Dataset-1	Position	0.21	0.76	0.36	0.67	0.39	0.77	0.29	0.72	0.40	0.72
	Velocity	0.38	0.31	0.53	0.29	0.62	0.41	0.48	0.21	0.59	0.40
Dataset-2	Position	0.26	0.84	0.25	0.88	0.32	0.83	0.21	0.84	0.27	0.85
	Velocity	0.52	0.36	0.60	0.25	0.63	0.33	0.44	0.31	0.53	0.35

Table 1. Prediction Results for Five Personality Traits using Principal Component Regression on both the datasets.

We took Principal Components of the features for the model against Linear Regression model to avoid overfitting. Table 1 contains the calculated RMSE and R^2 scores for individual personality traits from both the datasets after taking the Principal

Components. We repeated this experiment by varying the number of principal components. The results show comparatively inferior performance as compared to the Bayesian Regression.

In order to demonstrate the effect of varying components, Figure 1 contains the graph of R^2 and RMSE for the performed PCR on Openness from both Datasets.

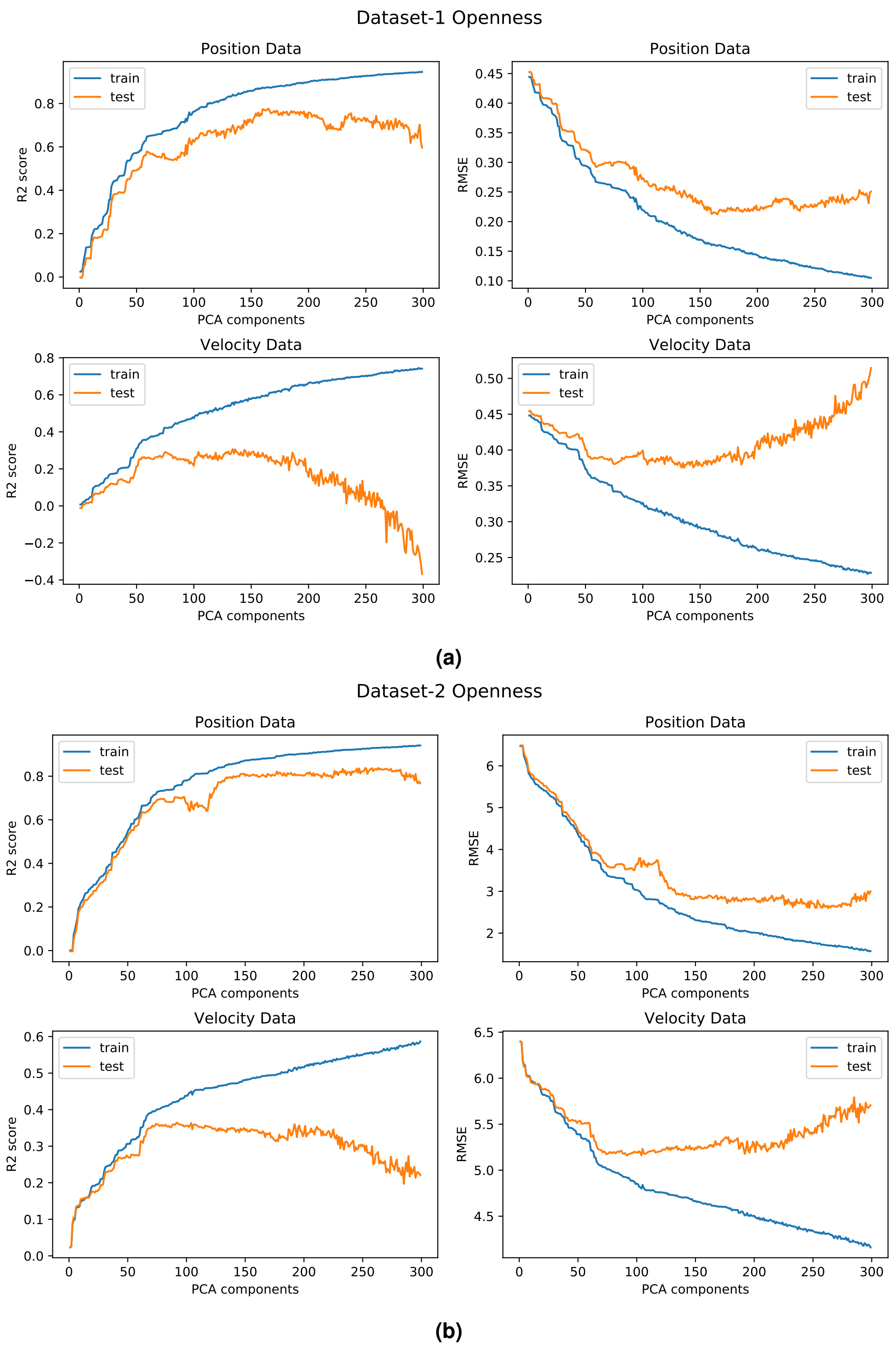

Figure 1. Principal Component Regression results on Openness personality trait from Dataset-1 and Dataset-2

Spearman Correlations between trait-wise personality across datasets.

	O_1	C_1	E_1	A_1	N_1	O_2	C_2	E_2	A_2	N_2
O_1	1.0					0.11				
C_1	-0.08	1.0				-0.17	0.73*			
E_1	-0.50	-0.61*	1.0			0.01	-0.63*	0.52		
A_1	0.29	-0.51	0.1	1.0		-0.08	-0.1	-0.06	0.32	
N_1	0.17	-0.14	-0.35	-0.29	1.0	0.35	0.01	-0.03	-0.12	-0.2
O_2	0.11					1.0				
C_2	-0.08	0.73*				-0.49	1.0			
E_2	-0.48	-0.43	0.52			-0.27	-0.24	1.0		
A_2	0.17	0.04	-0.10	0.32		-0.34	0.18	-0.56	1.0	
N_2	0.31	-0.24	0.23	0.03	-0.20	0.28	-0.57	-0.15	-0.23	1.0

$*p < 0.05$

Table 2. Results of the Spearman Correlation between the Mean-centered *Joint Importance Vectors* learned for personality traits.

Joint Importance Profile for Genre Prediction

Figure 2 displays the relative *Joint Importance* of Genre Preferences along with the mean plotted in each sub-figure.

Ward's Method

The distance between two clusters, A and B, using the ward's method is given by:

$$\Delta(A,B) = \sum_{i \in A \cup B} ||\vec{x}_i - \vec{m}_{A \cup B}||^2 - \sum_{i \in A} ||\vec{x}_i - \vec{m}_A||^2 - \sum_{i \in B} ||\vec{x}_i - \vec{m}_B||^2 \quad (4)$$

$$\Delta(A,B) = \frac{n_A n_B}{n_A + n_B} ||\vec{m}_A - \vec{m}_B||^2 \quad (5)$$

where $\vec{m}_j$, n_j represents the center of cluster, the number of points in cluster j. $\Delta(A,B)$ is called the merging cost of combining the clusters A and B. Ward's method keeps the merging cost as small as possible.

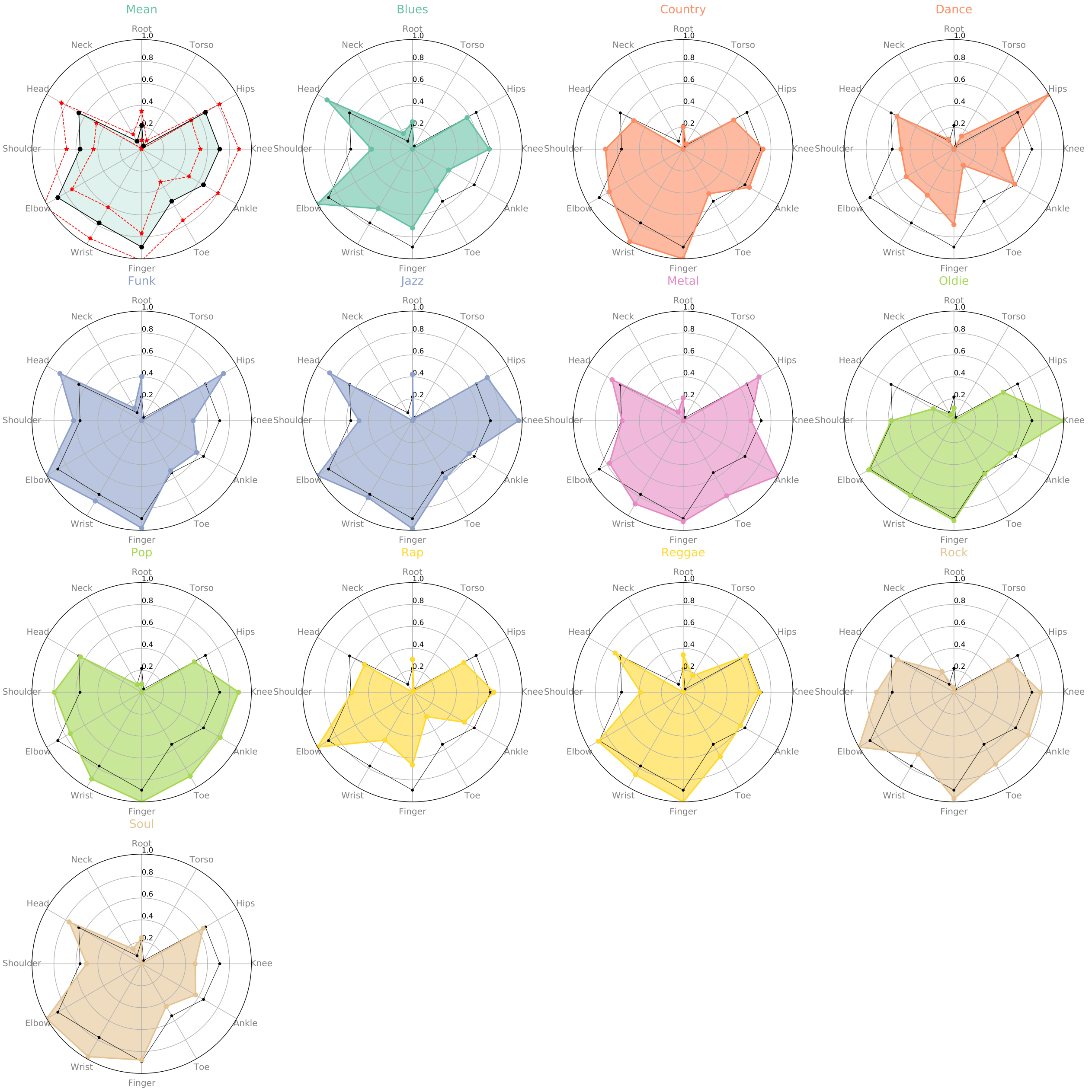

Figure 2. Relative importance of Joints of the 12 music preferences (Blues, Country, Dance, Funk, Jazz, Metal, Oldies, Pop, Rap, Reggae, Rock, and Soul.) using the Position Data. The black line indicates the mean importance of the corresponding joint marker. The red dotted line in the top left sub-figure indicates the standard deviation about the mean.
